# Differences, links, and roles of microbial and stoichiometric factors in microplastic distribution: A case study of five typical rice cropping regions in China

**DOI:** 10.3389/fmicb.2022.985239

**Published:** 2022-09-02

**Authors:** Yao Yao, Lili Wang, Lingxuan Gong, Gang Li, Weiming Xiu, Xiaomei Yang, Bingchang Tan, Jianning Zhao, Guilong Zhang

**Affiliations:** ^1^Agro-Environmental Protection Institute, Ministry of Agriculture and Rural Affairs, Tianjin, China; ^2^College of Natural Resources and Environment, Northwest A&F University, Yangling, China; ^3^Soil Physics and Land Management Group, Wageningen University & Research, Wageningen, Netherlands

**Keywords:** microplastics, microbiology, stoichiometric ratio, paddy soils, bacterial diversity

## Abstract

Microplastics (MPs), as new pollutants in agroecosystems, have already attracted widespread attention from scientists. However, our understanding of MP geographic distribution and its influencing factors across spatial scales remains poor. Here, a regional-scale field investigation was conducted to assess the distribution characteristic of MPs in five major rice-growing regions of China, and we explored the roles of biological and abiotic factors, especially stoichiometry and microbial influences on MP distribution. MPs were observed in all sampling sites, averaging 6,390 ± 2,031 items⋅kg^–1^. Sizes less than 0.5 mm and black and transparent MPs dominated. Fiber, classified as one of the MP shapes, occurred most frequently. MP community analysis, firstly used in paddy soil, revealed more black MPs abundance in Henan (HE), more rayon, blue, and other colors MPs in Hunan (HN), more transparent MPs in Tianjing (TJ), and more PE MPs in Heilongjiang (DB). Higher MP community diversity was found in most south paddy soils of this study, due to a broader range of sources. C/N showed a positive relationship with pellet-shaped MP abundance and MPs of size between 2 and 5 mm (*P* < 0.05). Chao1 index of soil microbial communities was positively correlated with the MP abundance, MPs of size less than 0.5 mm, and fiber abundance. The minimum temperature was positively correlated with MP abundance (*P* < 0.05), implying the potential effects of the freeze-thaw process might exist. The regression analysis highlighted the important role of population quantity in determining MP abundance (*R* = 0.421, *P* = 0.02). This study confirmed the wide distribution of MPs in different soil depths of paddy lands in China and demonstrated that its distribution was influenced by population quantity and environmental variables, such as microbiology. These findings could provide a basis for the toxicological behavior of MPs and the potential risk to human health.

## Introduction

Due to the extensive use and inappropriate disposal of plastic products, microplastic (MP) pollution has been recognized as a new threat to our earth systems and have become a research hotspot for scholars (Hu et al., 2019; Leifheit et al., 2021). Much evidence has verified the existence of MPs in oceans (Jiang Y. et al., 2020), fresh water (Miao et al., 2020), atmospheres (Dong et al., 2021), soils and other mediums (Abayomi et al., 2017; Bergmann et al., 2019; Wang et al., 2021). Polymer types, abundance, and distribution characteristics varied among different environments. Until now, most current studies focused on aquatic ecosystems. However, agroecosystems are a massive sink for MPs, with long-lasting presence and harmful effects. MPs could affect soil physicochemical properties, including soil bulk density, pH, and soil aggregates (Machado et al., 2018, 2019; Wang F. et al., 2020), and could even be toxic to microorganisms and soil animals (Jiang X. et al., 2020; Li et al., 2020). It has also been shown that MPs can accumulate in plants (Zhang H. et al., 2022), and are transferred through the food chain and food web, ultimately endangering human health (Huerta Lwanga et al., 2017; Wong et al., 2020). In addition, the release of additives in MPs (Li et al., 2020) and the adsorption of MPs to other agricultural soil pollutants such as heavy metals, antibiotics, and pesticides increase the risk of MPs (Dong et al., 2020b; Wang T. et al., 2020). Therefore, exploring the MP distribution in agricultural ecosystems is crucial to assess their ecological effects on farmland and human health.

To date, MP pollution has been serious in agricultural fields and many factors lead to its accumulation. For example, wind speed, precipitation, freeze-thaw cycling, and alternating wet and dry processes among different geographical locations can affect the migration, accumulation, and degradation of MPs (Wang et al., 2021; Yu et al., 2022; Zhang H. et al., 2022). Similarly, MP distribution could be influenced by soil properties. One study in Shouguang, Shandong Province, showed the correlation between soil texture and MP distribution, with sandy soils having more aeration pores, which facilitated MP transport, and loamy soils having tiny pores that facilitated long-term MP accumulations (Yu et al., 2021). Also, pH could affect MP distribution due to high pH inhibiting the adsorption between negatively charged MPs and soil, thus increasing their transport (Luo et al., 2020). Soil organic carbon (SOC) acted as a substrate to promote microbial degradation of MPs, the improvement of soil pore space caused by SOC could promote MP migration (Zhang S. et al., 2020; Guo et al., 2021). Furthermore, different regional land use patterns and crop types vary the use of mulch, tillage practices, and fertilizer application (Wang et al., 2021; Zhang H. et al., 2022), thus influencing the MP abundance. For instance, the pores produced by corn roots accelerated the MP movement (Guo et al., 2020; Li H. et al., 2021). Thus, exploring MPs among different agricultural soils is vitally essential for evaluating the risk of MPs to cropping systems. However, the role of activated carbon components [i.e., dissolved organic carbon (DOC)], microbial communities, and stoichiometric ratios (e.g., C/N, N/P) on MP distribution and their coupling relationship, remain largely unstudied.

Rice is an essential food crop with a large planting area, previous studies have revealed the adverse effects of MPs on rice germination, growth, and yield through metabolomics and transcriptomics (Zhang et al., 2021; Chen et al., 2022; Wu et al., 2022). Hence, investigating and studying the distribution characteristics of MPs in paddy soils is crucial for food security, plant and animal growth, and human health (Wang et al., 2021). Kim et al. (2021) found that MPs in Korean paddy soil of 0–5 cm depth were about 160 ± 92 items⋅kg^–1^. An investigation showed 16.1 ± 3.5 items⋅kg^–1^ MPs in paddy and duck rice fields (Lv et al., 2019). Existing studies also explored the effects of MPs on microorganisms, functional genes, greenhouse gas emissions, and migration and transformation of other heavy metals in rice paddy land (Dong et al., 2020a; Xiao et al., 2021; Han et al., 2022; Sun X. et al., 2022; Yang X. et al., 2022). However, the related previous researches are rather fragmented, and have a considerable discrepancy, while regional studies on the spatial partitioning characteristics of soil MPs and the influencing mechanisms in paddy soils are critically limited.

The properties of MPs are complex, including diverse shapes, colors, sizes, polymer types, and additives types (Rochman et al., 2019). Consequently, we should consider MPs as a collection of different pollutants rather than one pollutant when researching the source, fate, and impact of MPs on organisms and ecosystems (Rochman et al., 2019; Zhou et al., 2020). Recently, the new “MP communities” was proposed to solve this problem. MPs combined with various colors, shapes, and polymer types were regarded as MP communities, similar to microbial communities (Zhou et al., 2020, 2022; Li C. et al., 2021). MP communities could comprehensively describe MP characteristics and pollution and analyze the associations and differences in MPs in different environmental units or compartments, which is more conducive to the effective control of MP (Wang et al., 2019; Li C. et al., 2021; Zhou et al., 2022). Through integrated analysis, there is a particular link between MP communities in five environments (freshwater, seawater, freshwater sediment, sea sediment, and soil), which decreases with the increase in geographical location (Li C. et al., 2021). A study in the world’s third-largest river found MPs in different compartments were significantly district and highly correlated with geographical distance through MP community analysis (Yuan et al., 2022). Zhou et al. (2022) found that complex point and non-point sources in urban soils could cause the inhomogeneity and heterogeneity of MP communities.

China’s rice cropping areas span several climatic zones, resulting in multiple environmental factors and differences in the distribution of MPs. There is still a lack of knowledge on the distribution characteristics of MPs in different paddy soils, especially for the coupling relationship between MP distribution and soil properties, and meteorological conditions. Therefore, this study selected five major rice cropping areas in China. The objectives were to (1) determine and compare the abundance, distribution, and characteristics of MPs in different paddy soils and depths, and identify their potential sources; (2) apply the MP communities concept in paddy soils for the first time, and evaluate the differences and association of MP distribution, and provide a basis for MP risk assessment; (3) elucidate the coupling relationship among soil microbial communities, C/N, climatic conditions, and MP distribution and characteristics.

## Materials and methods

### Study site and sample collection

**FIGURE 1:**
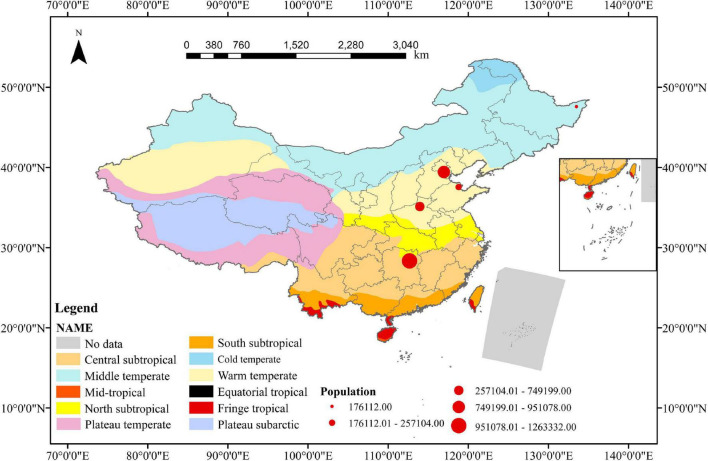
Soil sampling sites in this study.

Five major rice cropping regions in China were selected for this study. Specific locations are shown in Figure 1. The details of sampling sites were shown in Supplementary materials.

Soil samples were collected before the rice harvest in October 2021, and three plots (10 m × 10 m) were selected as three replicates for each sampling site. Using the S-shaped sampling method, 15 sampling points were selected for each plot. And 0–20 cm and 20–40 cm soil samples were taken, respectively with a 5 cm diameter soil auger. The soil samples from the same plot were mixed evenly to represent the entire sampling point. Stones and visible stubbles were removed and packed into aluminum boxes, and quickly brought back to the laboratory in ice boxes. Soil samples were divided into three parts, one part was stored at −80°C for microbial identification, one part was freeze-dried (Eyela freeze dryer, FDU-1200) for MP identification, and the remaining part was used for the determination of the soil physical and chemical properties.

### Microplastic extraction and identification

Microplastics were extracted from soil samples by density separation method, based on EPA and technical regulation for monitoring marine MP (Trial) (Masura et al., 2015) with modifications (Li et al., 2020; Wang et al., 2021; Zhang H. et al., 2022).

A 10 g lyophilized soil sample and 500 ml Zinc chloride solution (ZnCl_2_) were added to a 2 L beaker, stirred continuously for 48 h, then left to stand, transferred in batches to a partition funnel, then left to stand for 24 h. The upper liquid lay was collected and filtered through a 0.45 μm filter membrane and set aside. The flotation process was repeated three times as described above to ensure that all MPs were extracted. The material on the filter membrane was ultrasonically backwashed into a beaker, and the filter membrane was processed for microscopic examination to confirm that no MPs remained, then was placed in a heated temperature-controlled and speed-controlled shaker after adding Fenton reagent and hydrogen peroxide, and was then digested for 72 h. Then the solution was passed through a 0.45 μm filter membrane again.

Dried sample membranes were observed under a stereoscopic (Leica LAS X) microscope with a high-resolution digital camera and the number, size, shape, and color of MPs were all recorded. The polymer types of all MPs were identified using μ-FT-IR (is10, America). The spectra were measured in the range of 700–4,000 cm^–1^, with a resolution of 8 cm^–1^ and a scan count of 32. The test results were compared with a library of synthetic polymer spectra, and the sample composition was judged based on a match of more than 70%.

### Microplastic communities

This study applies the MP community concept in paddy soils and evaluates the differences and association of MP distribution.

Analysis of similarities (ANOSIM) and linear discriminant analysis (LDA) were used to compare differences between soil MP communities in the different geographic position; LDA effect size (LEfSe) was used to analyze the soil characteristic MP types in each sampling sites; LEfSe analysis is available on http://huttenhower.sph.harvard.edu/galaxy. The similarity of MP communities among different environments was tested by mantel based on Bray-Curtis distance. Previous studies have described the characteristics of MP pollution and speculated the source only through the single MP feature index, such as shape diversity index (Wang et al., 2019) and polymer type diversity index (Zhou et al., 2019). However, MPs are a group of contaminants aggregated with multiple properties, and it is not sufficient to describe them with a single diversity indicator (Zhou et al., 2022). Fully and comprehensively considering three diversity indices of color, size, and polymer type, a multiple composite index (MDII index) proposed by Li C. et al. (2021) was used to compare the diversity of MP communities and help in pollution source analysis.


$$\text{MDII}={\left(S\,i\,m\,p\,s\,o\,n\,\_\,s\,h\,a\,p\,e\times S\,i\,m\,p\,s\,o\,n\,\_\,c\,o\,l\,o\,r\times S\,i\,m\,p\,s\,o\,n\,\_\,p\,o\,l\,y\,m\,e\,r\right)}^{1/3}$$

### Determination of soil physical and chemical properties

In this study, we tested four activated carbon components: microbial biomass carbon (MBC), DOC, particulate organic carbon (POC), and permanganate oxidizable carbon (POXC) and the methods followed by Shen et al. (2021). MBC was fumigated with chloroform, extracted with potassium sulfate, and determined by a total organic carbon analyzer (Multi N/C3100, Hamburg, Germany). DOC was extracted using a water-soil ratio of 5:1, shaken at 25°C for 30 min, and centrifuged at 4,500 rpm for 20 min; the supernatant was passed through a 0.45 μm filter membrane and then analyzed by Multi N/C 3000 total organic carbon/total carbon analyzer (Multi N/C 3000, Hamburg, Germany). POC was extracted by (NaPO_3_)_6_ and then determined by SOC that failed to pass the 0.053 mm sieve. POXC was obtained by KMnO_4_ (333 mM) oxidation and calculated by its loss. C/N, C/P, and N/P were the ratio of total carbon to total nitrogen, total carbon to total phosphorus, and total nitrogen to total phosphorus, respectively.

### DNA extraction, amplification and sequencing of soil microorganisms

Soil microbiome DNA was extracted based on the instructions of the Power Soil DNA Isolation Kit (MO BIO Laboratories, Carlsbad, CA, United States). DNA mass and concentration was determined using a 1% agarose gel electrophoresis and spectrometer. The V3–V4 region of the bacterial 16srRNA gene was amplified using primers 338 F (5′-ACTCCTACGGGAGGCAGCAG-3′) and 806 R (5′-GGACTACNNGGGTATCTAAT-3′), and 8 bp of barcode sequence was added to each of the upstream and downstream 5′ primer ends to distinguish between the different samples. The PCR product was detected by a 1% agarose gel electrophoresis and purified with Agencourt AMPure XP nucleic acid purification kit. Then, the microbial diversity sequencing library was constructed and paired-end sequencing was performed using Illumina MiSeq PE300 high-throughput sequencing platform.

### Quality control

To avoid atmospheric and potential human contamination and ensure the accuracy of this experiment, we conducted strict quality control during the experiment by wearing masks, cotton lab coats, and gloves. All vessels were cleaned with ultrapure water and the cleanliness of each experimental apparatus was ensured. Two control experiments were set up to eliminate the influence of the control and the reagent, and a control spiking experiment with a recovery rate of 98–100% was also set up.

### Data analysis

All data were processed and analyzed using Excel 2013, SPSS 24, and R studio. Origin and R studio were used for graphical plotting. Duncan method in One-way ANOVA was used to compare the differences between distinct paddy soils, and an independent sample *T*-test was used to analyze the differences between different depths of soil in the same geographic position. The correlation between different MP indicators and environmental factors was tested using Spearman non-parametric method. The relative abundance of bacterial taxa (phylum) of different paddy soils was performed on Tutools platform https://www.cloudtutu.com. And the analysis of soil species in different soils was performed on Stamp software. The meteorological data were obtained from National Renewable Energy Laboratory. And the population quantity was obtained from http://www.citypopulation.de/.

## Results

### Soil chemical and microbial properties in different paddy soils

Soil properties varied among geographical locations and depths (Table 1). A higher C/N was found in the two soil layers of Shandong (SD), with 13.49 ± 1.06 in the topsoil layer (0–20 cm) and 14.52 ± 0.56 in subsoil (20–40 cm). The C/P and N/P of Hunan (HN) in the topsoil layer were the highest, with the value of 58.23 ± 1.70, and 4.86 ± 0.09, respectively.

**TABLE 1:** Soil properties of different paddy soils.

Type	Depth	pH	SOC	POXC	POC	C/N	C/P	N/P
SD	0–20 cm	7.92 ± 2.61Bb	9.40 ± 2.61Ca	3.45 ± 0.05Ca	3.97 ± 0.01Ca	13.49 ± 1.06Aa	13.52 ± 1.50Da	1.04 ± 0.02Ea
	20–40 cm	8.44 ± 0.03Aa	7.03 ± 0.47Eb	2.59 ± 0.37Eb	4.35 ± 0.66Ca	14.52 ± 0.56Aa	11.97 ± 0.82Da	0.82 ± 0.02Eb
HE	0–20 cm	7.83 ± 0.03Cb	9.82 ± 1.05Ca	2.76 ± 0.14Db	1.68 ± 0.20Ea	10.32 ± 0.99Ca	15.18 ± 1.47CDa	1.47 ± 0.02Da
	20–40 cm	7.88 ± 0.02Ca	8.50 ± 0.22Da	3.99 ± 0.46Da	1.95 ± 0.29Ea	10.54 ± 0.11Ca	13.17 ± 0.48Da	1.25 ± 0.04Db
TJ	0–20 cm	8.12 ± 0.02Ab	8.50 ± 0.52Ca	3.23 ± 0.19Cb	3.21 ± 0.33Da	9.31 ± 0.35Ca	16.87 ± 0.83Ca	1.81 ± 0.03Ca
	20–40 cm	8.37 ± 0.03Ba	10.46 ± 0.24Ca	4.61 ± 0.18Ca	3.41 ± 0.26Da	9.70 ± 0.14Da	16.76 ± 0.18Ca	1.73 ± 0.04Cb
HN	0–20 cm	6.91 ± 0.10Da	16.51 ± 0.55Ba	6.24 ± 0.05Aa	10.28 ± 0.40Ba	11.99 ± 0.19Ba	58.23 ± 1.70Aa	4.86 ± 0.09Aa
	20–40 cm	6.55 ± 0.06Db	15.54 ± 0.47Bb	6.13 ± 0.32Ba	11.52 ± 1.00Aa	11.90 ± 0.28Ba	51.51 ± 2.36Ab	4.33 ± 0.11Ab
DB	0–20 cm	5.49 ± 0.06Ea	18.81 ± 0.84Ab	4.58 ± 0.61Bb	15.41 ± 0.01Aa	9.82 ± 0.25Ca	34.09 ± 1.34Ba	3.47 ± 0.05Bb
	20–40 cm	5.46 ± 0.02Ea	19.20 ± 0.65Aa	7.61 ± 0.15Aa	7.01 ± 0.40Bb	9.33 ± 0.28Db	33.95 ± 1.20Bb	3.64 ± 0.03Bb

SOC, soil organic carbon, g^●^kg^–1^; DOC, dissolved organic carbon, mg^●^kg^–1^; MBC, microbial biomass carbon, mg^●^kg^–1^; POXC, permanganate oxidizable carbon, g^●^kg^–1^; POC, particulate organic carbon, g^●^kg^–1^; C/N, ratio of total carbon to total nitrogen; C/P, ratio of total carbon to total phosphorus; N/P, ratio of total nitrogen to total phosphorus. Different capital letters indicate the significant difference among sampling sites, and different lowercase letters indicate the significant differences between soil depths (*P* < 0.05) (*n* = 3).

**FIGURE 2:**
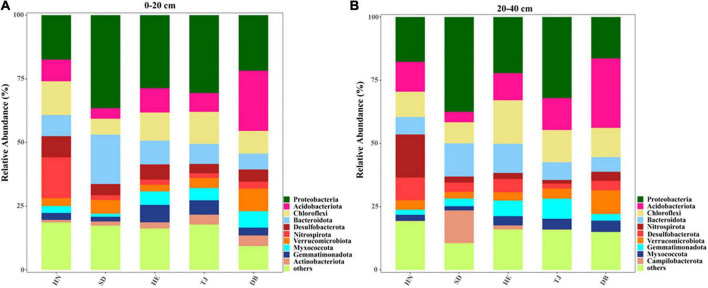
The relative abundance of bacterial taxa (phylum) in different paddy soils; **(A)** 0–20 cm; **(B)** 20–40 cm.

The microbial diversity index Chao1 in the topsoil of the Tianjing (TJ) site showed lower values than that of other types at the topsoil. At the subsoil layer, HN had higher Chao1 than that in TJ and Heilongjiang (DB) soils. Additionally, we further analyzed the core microbes at two soil layers of the five sites (Figure 2). At the phylum level, the bacterial compositions of five soils were dominated by *Acidobacteriota*, *Chloroflexi*, *Proteobacteria*, *Bacteroidota*, and *Nitrospirota*. The abundance of *Nitrospirota* was significantly higher in HN, whereas the abundance of *Proteobacteria* in HN was significantly lower than that in SD (Supplementary Figure 1). Also, the highest abundance of *Bacteroidota* was found in SD (Supplementary Figure 1). Moreover, DB in subsoil had a higher abundance of *Myxococcota* than SD and HN, while SD had a lower abundance of *Myxococcota* than TJ (Supplementary Figure 1). The highest abundance of *Nitrospirota* and *Verrucomicro* in subsoil layers were found in HN and DB, respectively (Supplementary Figure 1).

### Microplastic properties in different paddy soils

**FIGURE 3:**
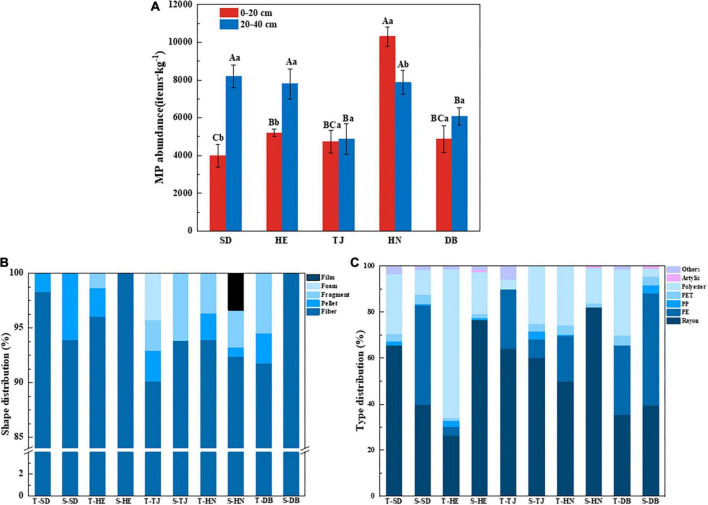
Microplastic (MP) abundance in different paddy soils **(A)**; percentage of MP shape distribution **(B)** and compositions **(C)** of paddy soils among different geographic positions. T-: 0–20 cm layer; S-: 20–40 cm layer. Different capital letters indicate the significant difference among different geographic positions, and different lowercase letters indicate the significant differences between soil depths (*P* < 0.05) (*n* = 3).

Microplastics were found in all sampling sites, with an average of 6,390 ± 2,031 items⋅kg^–1^. Geographical position, soil layers, and interaction significantly affected MP abundance (Supplementary Table 1; *P* < 0.05). The highest and lowest MP abundance in all samples were measured in topsoil layers of HN and SD, with the value of 10,300 items⋅kg^–1^ and 4,000 items⋅kg^–1^, respectively. In subsoil layers, SD, Henan (HE), and HN had higher MP abundance than TJ and DB (Figure 3A; *P* < 0.05). In topsoil layers, HN had higher MP abundance than others, while SD had lower MP abundance than others. MP abundance in the topsoil layers of HN was significantly higher than that of subsoil layers, while MP abundance in topsoil layers of SD and HE was significantly lower than that of subsoil layers (Figure 3A; *P* < 0.05).

The size distribution of MPs is shown in Supplementary Figures 2A,B, and was divided into <0.5 mm, 0.5–1 mm, 1–2 mm, and 2–5 mm. Overall, the proportion of MPs less than 0.5 mm at all the sampling sites was the largest, followed by 0.5–1 mm. In regards to different sampling sites, the abundance of particle size less than 0.5 mm showed the highest concentration (60.89%) in the topsoil of HN. The subsoil layer of TJ had significantly higher particles (30.67%) ranging from 0.5 to 1 mm than those of others. The MPs sized between 2 and 5 mm showed the highest concentration in the topsoil layer of SD (33.95%) (Supplementary Figures 2C–F; *P* < 0.05).

**FIGURE 4:**
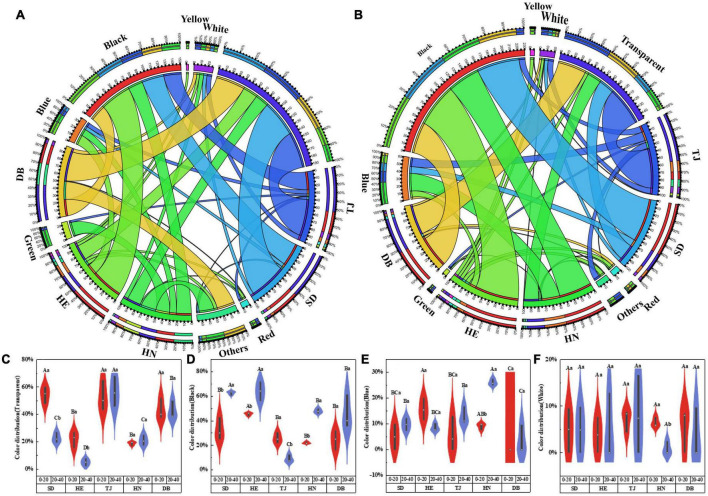
Color **(A)** in 0–20 cm, color **(B)** in 20–40 cm distribution of MPs in different paddy soils; violin plots showing the variance analysis of four colors: transparent **(C)**; black **(D)**; blue **(E)**; white **(F)**. Different capital letters indicate the significant difference among different geographic positions, and different lowercase letters indicate the significant differences between soil depths (*P* < 0.05) (*n* = 3).

Microplastics were categorized by color as white, black, transparent, blue, and other colors. Transparent and black were the most common colors in all samples, accounting for 2.6–67.86%, and 7.1–71.79%, respectively (Figures 4A,B). The abundance of black MPs in the subsoil layer of SD and DB was higher than those in other soils, and the abundance of transparent MPs was the lowest in the subsoil layer of DB (Figures 4C–F; *P* < 0.05).

Particle shapes were categorized as fiber, pellet, fragment, foam, and film in all samples, with the shape and composition of MP in different soil types and layers varying greatly. As shown in Figure 3B, fiber was the most common shape in paddy soils, accounting for 91.8–100%. There was only fiber in the subsoil layers of HE and DB. Overall, the topsoil layer had more abundant MP shapes than those of the subsoil layers in this study. MPs shaped like foam and film were only found in the topsoil layer of TJ and subsoil in HN, respectively.

Nine types of polymers were identified from all sampling sites, which were Rayon, polyethylene (PE), polypropylene (PP), PP + PE, polyethylene terephthalate (PET), and Polyester, Acrylic, polystyrene (PS), and PA (nylon) (Figure 3C). Among them, rayon was dominant, accounting for 26.58–82.05%. Polyester was the second most abundant type of MPs, accounting for 3.49–64.57%, of which the topsoil layer of HE had the highest polyester concentration. Additionally, DB had higher PE than other sites.

### Microplastic community characteristics in different paddy soils

**FIGURE 5:**
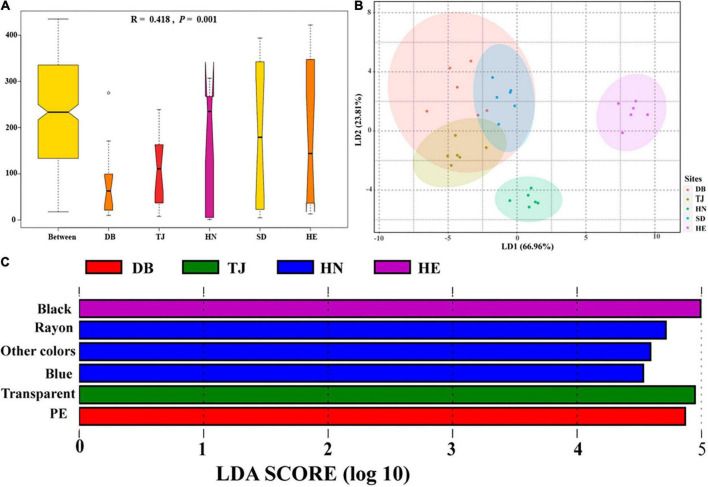
Differences of microplastic communities based on shape, color, and polymer types in different environments. Analysis of similarities (ANOSIM) was used for variance testing, and *y*-axis represents the dissimilarity ranks between and within environments, **(A)**; Linear discriminant analysis (LDA) was used to maximize the differences of paddy soils among geographic positions, **(B)**; LDA Effect Size (LEfSe) was used to identify characteristic microplastic types of paddy soils among geographic positions, **(C)**.

Analysis of similarities showed that the effect of geographical position on the composition of the MP communities was significantly greater than that of soil layers (Figure 5A; *R* = 0.418, *P* = 0.001). LDA axes1 and LDA axes2 explained the differences of 66.96 and 23.81% for MP communities among different locations, respectively (Figure 5B). HE and HN were significantly different from the other communities, but the MP communities of SD, DB, and TJ were not clearly distinguished on the LD1 and LD2 axes. LEfSe analysis was used to find the significantly discriminant MP types in each soil (Figure 5C). We found that there were more black MP abundances in HE, more rayon, blue, and other colors’ MPs in HN, more transparent MPs in TJ, and more PE MPs in DB. Each respective soil type has a characteristic MP type, except for SD.

In addition, we analyzed the similarity of MP communities by Mantel test based on Bray-Curtis distance (Supplementary Figure 4A). There was a certain positive correlation between MP communities of different locations, with the coefficient R between 0.38 and 0.86. The correlation coefficient between TJ and HE, as well as between DB, SD, and HE, as well as between TJ and SD were significant. MDII index was used to reflect the composition of MP communities, indicating the number of pollution sources. In this study, we found that HN and DB had higher MP community diversity, implying that these two soils had a wider range of MP sources (Supplementary Figure 4B).

### Correlation analysis among soil properties, meteorological conditions and microplastic characteristics

**FIGURE 6:**
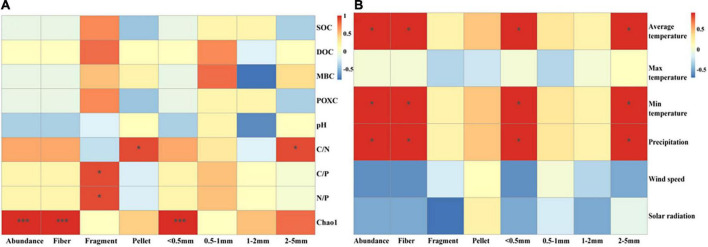
Linkage of main shape and size MP abundance with soil properties **(A)** and meteorological factors **(B)**. Significant differences of paddy soils among geographic positions were indicated by *P < 0.05, **P < 0.01, ***P < 0.001.

**FIGURE 7:**
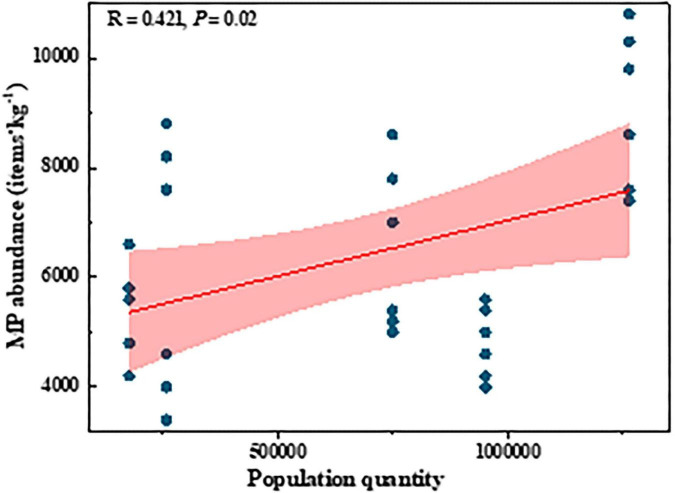
Linear correlations between MP abundance and population quantity.

Microplastic abundance of both pellet shape and size between 2 and 5 mm, positively correlated with C/N (Figure 6A). The abundance of fragment-shaped MPs, positively correlated with C/P as well as N/P (Figure 6A). There was an extremely significantly positive correlation between the Chao1 index and MP abundance, fiber, and size less than 0.5 mm (Figure 6A). Furthermore, we also analyzed the relationship between meteorological conditions and MP distribution characteristics (Figure 6B). The abundance of dominant MP shapes and sizes was proportional to average temperature, minimum temperature, and precipitation. There was a significantly positive correlation between MP abundance and average temperature, minimum temperature, and precipitation. Interestingly, we found solar radiation was inversely proportionated to MP abundance, fiber, fragment, pellet, and size less than 0.5 mm in top soil layers (Supplementary Figure 5). Additionally, positive relationships between population quantities and MP abundance were observed (Figure 7).

## Discussion

### The occurrence of microplastics in paddy soils as affected by human activities

Firsthand evidence of the MP pollution characteristics in the soils along different geographical locations and soil layers is thoroughly explored in this study. MPs were detected in all the paddy land sites from the north to the south of China (Figure 3A), indicating the severity and prevalence of MP pollution. The concentration of MPs ranges from 4,000 to 10,300 items⋅kg^–1^ (Figure 3A), which is comparable to those in previous studies, such as 320–12,560 items⋅kg^–1^ in vegetable farmland of Wuhan (Chen et al., 2020), and 900–40,800 items⋅kg^–1^ in cultivated soil of Yunnan in China (Huang B. et al., 2021). Zhou et al. (2019) reported the MP abundance ranged from 43,000 to 6,20,000 items⋅kg^–1^ in Wuhan vegetable patches, which was much higher than our results. This may be because the Wuhan vegetable patches were located near an industrial area with various pollution sources (Zhou et al., 2019). In addition, the MP abundance in our study was much higher than other results, such as a concentration of 0–260 items⋅kg^–1^ in the greenhouse soil of Qinghai Tibet (Feng et al., 2021) and 240–3,660 items⋅kg^–1^ in the farmland of Qinghai (Lang et al., 2022). This is because Qinghai is far from industrial and commercial areas, the high population density, complex pollution sources, and frequent human activities (Lang et al., 2022; Yang L. et al., 2022) leading to a higher MP abundance in our study area.

Overall, these studies showed that MPs were widespread in various agricultural soils and their abundance fluctuated widely. The variation of MP abundance in different agricultural areas may depend on numerous factors, such as sewage irrigation, pollution source, crop type, tillage practices, fertilizer application, mulch use, and so on (Ding et al., 2020; Lang et al., 2022). Also, in our study, divergent differences were found in MP abundance among different paddy lands, and MP abundance in the topsoil layer of HN was significantly higher than that in other soils (Figure 3A). This might be due to: (1) The HN sampling sites were surrounded by a higher population quantity (Figure 1) which may have frequent human activities; (2) The source of irrigation water in the HN rice-growing area is from a tributary of the Xiangjiang River, which has been reported to be rich in MPs in its water and sediment (Wen et al., 2018; Yin et al., 2019); (3) HN rice is grown over two seasons per years, and the amount and frequency of irrigation were higher than those in the other regions of this study. Previous studies found that irrigation source, frequency, and volume may contribute to the differences in MP abundance of various agricultural sources (Wang et al., 2021).

Microplastics in the soil come from a wide variety of sources, including agricultural activities, atmospheric deposition, irrigation water, sludge application, and roads (Sommer et al., 2018; Bergmann et al., 2019; Ding et al., 2020; Corradini et al., 2021). The color, shape, and polymer type of MPs may be indicative of the potential sources to some extent. Fibers were the dominant shape in paddy soils of our study (Figure 3B). Fibers may come from fishing nets, fishing line, rope breakage, and synthetic laundry (Abayomi et al., 2017; Mahon et al., 2017; Zhang X. et al., 2020), and were widely found in the Yongjiang River (Zhang X. et al., 2020), Xiangjiang River (Yin et al., 2019), Pearl River (Lin et al., 2018), as well as Yangtze River (Wang et al., 2017). A large number of fibers in paddy soils may have originated from irrigation water, synthetic fibers have also been used as indicators of wastewater sludge (Corradini et al., 2021). Although the irrigation water of DB and HE was groundwater with lower MP abundance, it may become contaminated with MPs while flowing from the irrigation source to the farm (Bläsing and Amelung, 2018), resulting in fiber MP pollution. Pellets were found in all topsoil layers among the different geographic positions. Pellets mainly originated from personal care products such as toothpaste and face wash in domestic wastewaters (Isobe, 2016), implying the source of MPs in paddy soil potentially originated from these irrigated waters. The foam was only found in the topsoil of TJ (Figure 3B), which may be due to TJ’s proximity to the Bohai Sea, frequent fishing activities generated foam (Xu et al., 2021), and the small density of foam made it difficult to migrate downward and it stayed in the surface layer. Fragment and film were found in paddy soils, mostly from agricultural tools, plastic packaging material, and woven bags (Antunes et al., 2013; Cao et al., 2021). Moreover, transparent and black MPs accounted for the majority of MPs in our study (Figures 4C,D). Transparent MPs usually come from plastic bags and plastic film (Chen et al., 2020; Wang et al., 2021), and they could also be caused by long-term weathering and discoloration of colored MPs (Zhu, 2015). Other colors of MPs may come from colored bags and colored laundry (Browne et al., 2011; Wen et al., 2018), and are easily predated by other organisms (Hoarau et al., 2014), resulting in lower abundance. MPs less than 0.5 mm accounted for the largest proportion in our study (Supplementary Figures 2A,B), indicating tillage and agricultural machinery accelerated the degradation of MPs (Yu et al., 2021).

In addition, MDII index was proposed to reflect MP community diversity and indicate pollution complexity (Li C. et al., 2021). Richer colors, shapes, and polymer types may have more uniform contamination sources (Wang et al., 2019; Li C. et al., 2021; Zhu et al., 2021). The MDII index of HN was significantly larger than that of SD and TJ, indicating the sources of pollution in HN with higher population quantities were more extensive and each source contributed equally.

### Effect of soil microbial communities and soil stoichiometry on microplastic distribution in paddy soils

It is well known that soil properties can affect the migration and transformation of MPs, while MPs can also affect soil properties (Qian et al., 2021; Sajjad et al., 2022; Zhang H. et al., 2022; Zhang J. et al., 2022). There are strong interactions between soil microorganisms and MPs. MPs could provide habitats for microorganisms (Miao et al., 2020), but MPs are selective and only promote the growth of specific microorganisms (Li et al., 2020). In addition, some microorganisms can use MPs as a carbon source (Seeley et al., 2020), both to provide energy for their growth and to promote the degradation of MPs through secreted oxidoreductases and hydrolases (Sudhakar et al., 2008). Furthermore, the degradation process of MPs may produce toxic substances such as DPB, affecting the growth of microorganisms (Wang et al., 2018). However, the coupling relationship between MPs and microorganisms is still limited, especially in the paddy soils of China. The Chao1 index is commonly used as a diversity index to indicate the abundance of microbial species. Our study found a significantly positive correlation between Chao1 and MP abundance (Figure 6A). On the one hand, MPs could provide habitats and a carbon source to microorganisms and promote their growth. On the other hand, microorganisms increased MP abundance and the percentage of less than 0.5 mm MPs by aging large pieces of plastics and MPs. Moreover, we revealed a significantly positive correlation between Chao1 and fiber abundance. This is because the large surface area and rich adsorption sites of fiber could facilitate microorganism attachment and growth (Rillig et al., 2017a; Dong et al., 2021), and the shape of fiber could increase the porosity and aeration of soil to promote growth (Wan et al., 2019). Analysis of soil microbial community composition revealed the dominant species in paddy soils including *Acidobacteriota*, *Chloroflexi*, *Proteobacteria*, *Bacteroidota*, and *Nitrospirota* (Figures 2A,B). Previous studies also confirmed the ability of MPs to promote the growth of *Proteobacteria*, *Bacteroidota*, *Actinobacteria*, *Acidobacteriota*, etc. (Qian et al., 2018; Huang et al., 2019; Qi et al., 2020). *Proteobacteria* was reported to be related to the aging of refractory polymers such as MPs (Yan et al., 2021).

Interestingly, we found soil MP abundance varied inconsistently with the two soil layers of the sampling sites (Figure 3A). MP abundance in SD and HE increased with soil depth, while MP abundance in HN decreased with soil depth, and the MP abundance in DB did not show significant variance between different depths. Some previous studies reported tilling may result in a uniform distribution of MPs under a vertical gradient (Harms et al., 2021), while others detected MP abundance decreased with the increase of depths due to the activity of earthworms (Harms et al., 2021). The variation of MPs at different soil depths may be influenced by various factors such as microorganisms, soil porosity, agricultural practices, protozoa, plant roots, fungi, etc. (Rillig et al., 2017b; Bläsing and Amelung, 2018; Cao et al., 2021; Zhang H. et al., 2022), contributing to the vertical variation of MPs in different paddy soils of this study. Additionally, the clayey texture and tiny porosity of the soil in HN (Liu et al., 2018) prevented MPs from moving down from the surface layer, resulting in a higher abundance of soil MP in topsoil than in subsoil.

The concentration and ratio of C, N, and P could be considered the main drivers of microbial diversity and organic carbon decomposition (Bradshaw et al., 2012; Guo and Jiang, 2019). Previous studies also revealed N and P could promote microbial growth and release oxidoreductase and hydrolase increasing soil fertility, thus effectively degrading MPs (Zhang H. et al., 2022). We found that C/N was positively associated with size 2–5 mm MPs and pellets. The high C/N suppressed the decomposition of organic matter and the abundance of microbes (Cleveland and Liptzin, 2007), leading to the accumulation of 2–5 mm MPs. Unlike other MP shapes, pellets can bind with soil particles and did not reduce soil capacity (Sun Y. et al., 2022). *Proteobacteria* which could age MPs preferred to settlement in low capacitance soils (Sun Y. et al., 2022). Likewise, N/P and C/P positively related with fragments. Another study found *Gemmatimonadota* and *Proteobacteria* with degradable MPs (Zhang S. et al., 2020; Yan et al., 2021; Yi et al., 2021) were strongly inversely associated with N/P (Delgado-Baquerizo et al., 2017), which also explains our result. Furthermore, the C, N, and P contained in MPs and their additives (De Souza Machado et al., 2019) also affected the stoichiometric ratio of the soil, enhancing the correlation between them. Overall, there is still a large gap in the study of the effect of soil stoichiometric ratio on MP characteristics and their mechanism, with more in-depth research needed in the future.

### Effect of meteorological conditions on the microplastic distribution in paddy soils

This study monitored MP communities in paddy soils from the North to the South of China, with average temperatures ranging from 2.9 to 16.8°C. The abundance, shape, size, and polymer type of soil MP differed significantly along geographical position. This may be caused by pollution sources, population scale, human activity intensity, soil properties, and meteorological conditions (Li C. et al., 2021; Yang L. et al., 2022; Zhang H. et al., 2022). The characteristic MPs of HN were rayon and blue (Figure 5C), which mainly come from the wastewater from synthetic clothing washing (Xu et al., 2021), as well as higher temperatures and more frequent irrigation activities. The characteristic MPs in TJ were transparent (Figure 5C), indicating a high degree of MP weathering in the TJ area (Zhu, 2015). Furthermore, large quantities of PE were found in DB (Figure 5C), which may be due to the use of plastic film made of PE (Kim et al., 2021) due to lower temperature conditions in the northeast parts of China.

Furthermore, MP communities in different paddy soils were found to be correlated through the Mantel test based on Bray-Curtis distance in this study (Figure 4A). The possible reasons are: (1) They may have similar contamination sources (Li C. et al., 2021); (2) MP could be transported by the atmosphere as well as surface runoff (Dong et al., 2021; Zhang H. et al., 2022). MP communities in SD, TJ, and HE were significantly correlated because soil properties and meteorological conditions were similar in the same climatic zones. These correlations suggested that MPs may move between different geographic positions, providing direct evidence of MP cycling (Bank and Hansson, 2019; Rochman and Hoellein, 2020).

In our study, the monthly mean temperature was positively related to MP abundance, fiber, MPs with size less than 0.5 mm, as well as MPs with size between 2 and 5 mm (Figure 6B). The highest average temperature was found in HN, and the highest MP abundance was also found in HN. Elevated temperatures promoted the aging of large plastic and low molecules through physical and chemical effects (Qian et al., 2021). Our results are supported by Zhang’s findings, which reported a positive 0 cm ground temperature dependence of MP abundance in the Qinghai-Tibet Plateau (Zhang H. et al., 2022). The suitable temperature could promote the growth of microorganisms, thus accelerating the aging of large pieces of plastic and MPs, increasing the abundance of MPs and MPs with sizes less than 0.5 mm and 2–5 mm. Moreover, there was a significantly positive correlation between MP abundance and precipitation. The presence of MPs in the atmosphere has been widely demonstrated and they could enter terrestrial ecosystems through rainfall (Dong et al., 2021; Huang Y. et al., 2021). Research showed that precipitation was a positive driver of atmospheric MP deposition and contributed to MP accumulation on Qinghai-Tibet Plateau (Yang L. et al., 2022; Zhang H. et al., 2022). Another study also found that up to 21.9 g/L of MPs in atmospheric sediment, contributed to most of the MP entering Nam Co Lake (Dong et al., 2021). Hence, MPs may be present in the atmosphere and accumulate in the soil during precipitation.

Moreover, we also found MP abundance in 0–20 cm layer was inversely correlated with solar radiation (Supplementary Figure 5). Previous studies concluded that stronger solar radiation and ultraviolet light can promote decomposition and increase MP abundance (Liu et al., 2014; Singh, 2015; Feng et al., 2021), which is inconsistent with our results. This indicated that the effect of solar radiation on MPs was limited in this study. It might be due to that the refraction and reflection of UV light by water in paddy soil weaken the effect of solar radiation on MPs. HN is in a subtropical monsoon climate, which is wet and rainy, and has many clouds causing solar radiation intensity to break. The high density of rice cultivation also hinders the aging effect of UV radiation from the sun. Additionally, we found wind speed was inversely correlated with MP abundance, which is inconsistent with another study in which a positive correlation between MPs and wind speed was found. This may be due to high-density rice cultivation (Wang et al., 2021) and the long-term flooding state of paddy soil weakening the effect of wind speed on MP transport.

Another amazing finding was that the minimum temperature in our study showed a positive correlation between MPs and MPs with sizes less than 0.5 mm and 2–5 mm, which is most likely due to the alteration of the freeze-thaw process. Previous studies found that freeze-thaw accelerated the aging and degradation of MPs, reduced their particle size, increased their adsorption capacity, and changed their molecular chemical structure through variations in moisture and surface heat balance (Chen et al., 2021). During the freezing period, the soil was cold, shaded, and anoxic, decreasing the rate of MP aging, while during the thawing period, the soil acted as an MP sink releasing MPs and accelerating their aging process (Chen et al., 2021; Dong et al., 2021). Thus, when the minimum temperature rose, it could expedite the melting of the permafrost later and the release of MPs, and also promote the large pieces of plastic and MP degradation. This verifies the results of our study that DB with the lowest minimum temperature had lower MP abundance.

## Conclusion

In this study, we conducted a comprehensive study to investigate the geographical distribution patterns and impact factors of MPs in the paddy field at regional scales. Overall, MPs were widely distributed in the soils, fiber-shaped and size less than 0.5 mm MPs dominated, implying that paddy soils are heavily polluted with MPs. We proposed that irrigation might be the main potential MP source. Furthermore, planting patterns, soil physicochemical properties, meteorological conditions, and population quantity were verified to affect the transport, accumulation, and fragmentation of MPs in paddy soils. Our findings fill the gap in our understanding of the distribution characteristics and influencing factors of MPs in paddy soils from the North to the South of China In the future, more attention was highly expected to be exerted into the toxicological behavior of MPs in rice paddy field and the potential transport of MPs to the human food chain.

## Data availability statement

The datasets presented in this study can be found in online repositories. The names of the repository/repositories and accession number(s) can be found below: NCBI SRA, PRJNA858258.

## Author contributions

YY: investigation, data curation, visualization, and writing – original draft preparation. LW: conceptualization, supervision, writing – review and editing, and funding acquisition. LG and WX: visualization. GL: methodology and data curation. XY, BT, and JZ: review and editing. GZ: project administration. All authors contributed to the article and approved the submitted version.
