# Supplementary material for: Differences, links, and roles of microbial and stoichiometric factors in microplastic distribution: A case study of five typical rice cropping regions in China
Source: Front Microbiol. 2022 Sep 2;13:985239. doi: 10.3389/fmicb.2022.985239 (PMC9478377; doi:10.3389/fmicb.2022.985239)
Supplement: Supplementary file 1 [file Data_Sheet_1.docx]

Supplementary Materials

**The details of sampling sites.**

Tongjiang country (DB) is located in northeastern Heilongjiang Province, China, and belongs to the middle temperate humid climate zone, with an average annual temperature of 2.9 ℃, average annual precipitation of 532.7 mm, a soil type of Eutric Cambisols, a vegetation type of annual grain crops and hardy cash crops, a population quantity of about 176,112 people, and a low intensity of human activity. Yuanyang country (HE) is located in northern part of Henan Province, China, and has a warm temperate continental monsoon climate with an average annual temperature of 14 ℃, average annual precipitation of 573.4 mm, Calcaric Fluvisols soil type, and a rice and wheat crop rotation, with a population quantity of about 749,199 people, and a slightly higher intensity of human activity. Dongying country (SD) is located in northern part of Shandong Province, China, and has a warm temperate continental monsoon climate with an average annual temperature of 12.8 °C, average annual precipitation of 555.9 mm, a soil type of Haplic Solonchaks, rice and wheat crop rotation, with a population quantity of about 257,104 people and a lower intensity of human activity. Wuqing District (TJ) is located Northwest of Tianjin, China, and has a warm temperate semi-humid continental monsoon climate with an average annual temperature of 11.6 °C, average annual precipitation of 606 mm, a Cumulic Anthrosols soil type, rice, and wheat crop rotation, with a population quantity of about 951, 078 people and a slightly higher human activity intensity. Ningxiang (HN) is located in northeastern part of Hunan Province, China, with a subtropical continental monsoonal humid climate, an average annual temperature of 16.8 °C, average annual precipitation of 1358.3 mm, a soil type of white pulpy soil, rice cultivation for two seasons per year, with a population quantity of about 1, 263, 332 people and high intensity of human activities. The above data were obtained from China Statistical Yearbook 2021.

**Supplementary Information Captions**

**Supplementary Figure 1** The bacterial taxa (phylum) showing significant differences of different paddy soils in 0-20 cm.

**Supplementary Figure 2** Size (A) in 0-20 cm, size (B) in 20-40 cm distribution of MPs in different paddy soils; violin plots showing the variance analysis of four sizes: <0.5 mm (C); 0.5-1 mm (D); 1-2 mm (E); 2-5 mm (F); Different capital letters indicate the significant difference among geographic position, and different lowercase letters indicate the significant differences between soil depths (*P*< 0.05) (n = 3).

**Supplementary Figure 3** Microscopic images of four types of microplastics: fiber (A); pellet (B); film (C); fragment (D).

**Supplementary Figure 4** Mantel test based on Bray-Curtis distance, (A); the MDII index of paddy soils among different geographic positions, (B). Different lowercase letters indicate different levels (*P* < 0.05). Significant differences of paddy soils among different geographic positions were indicated by **P <* 0.05*, **P <* 0.01*, ***P <* 0.001*.*

**Supplementary Figure 5** Linkage of main shape and size MP abundance with meteorological factors in 0-20 cm. Significant differences of distinct paddy soils were indicated by **P <* 0.05*, **P <* 0.01*, ***P <* 0.001.

**Supplementary Table 1** Two-way ANOVA of MP abundance in different paddy soil layers among geographic positions

**
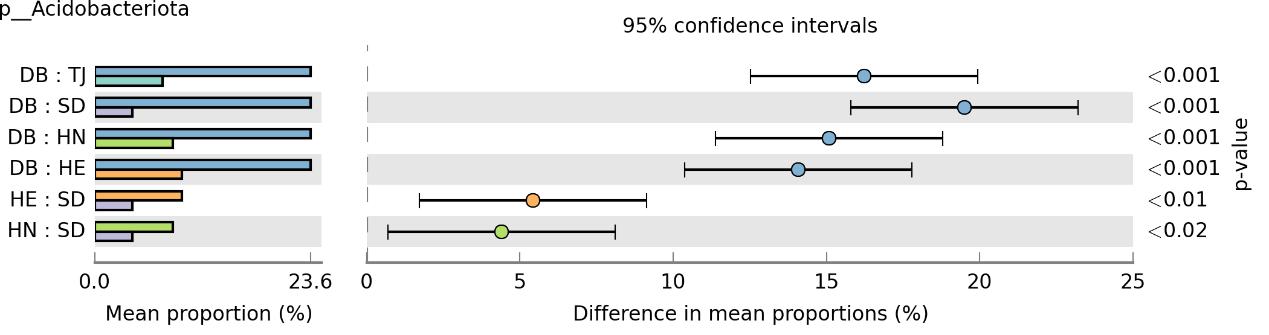

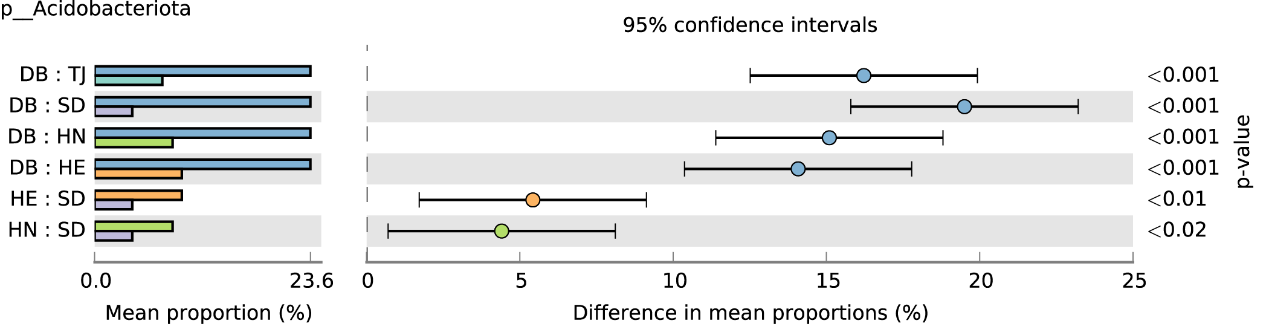

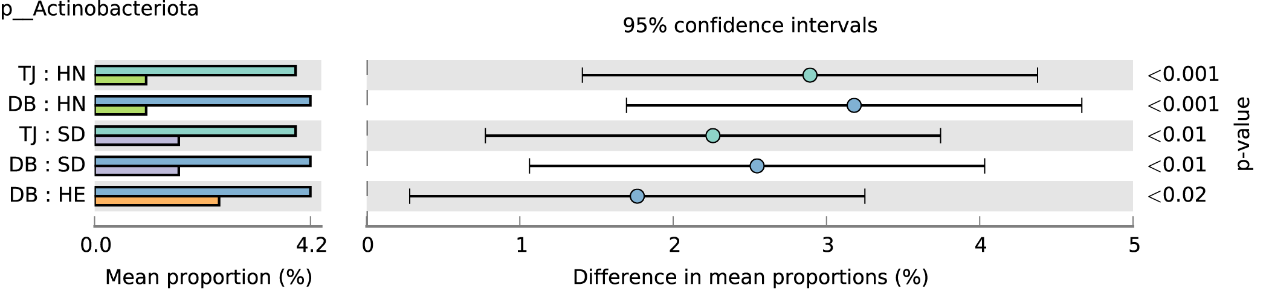

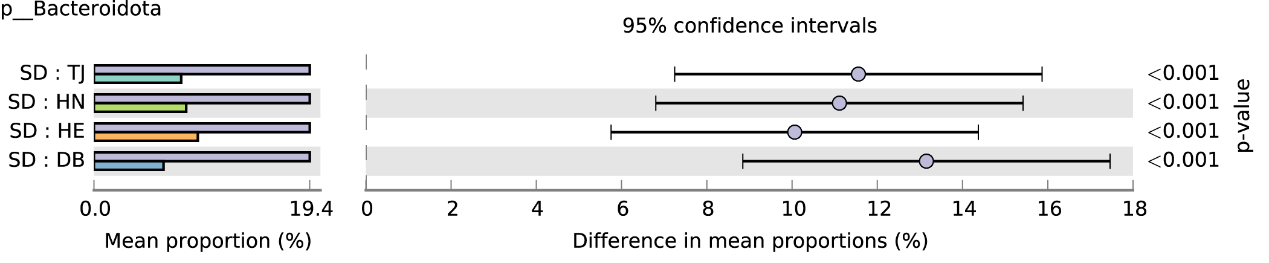

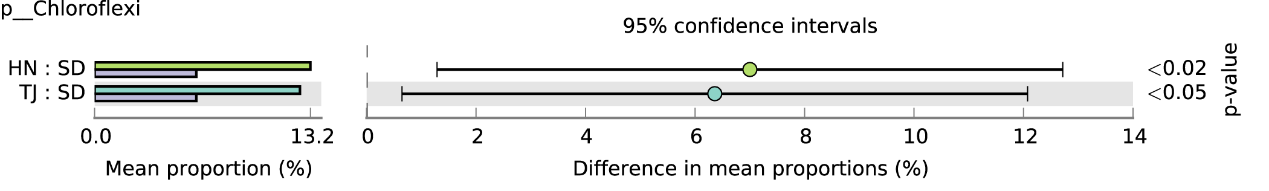

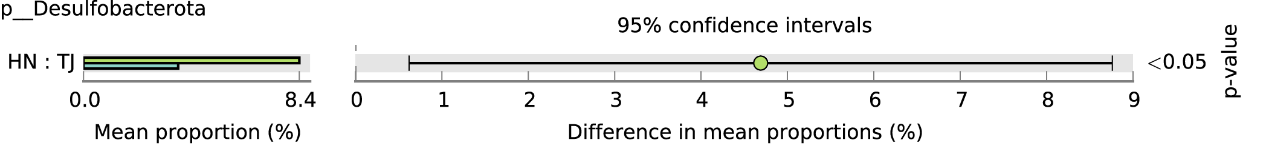

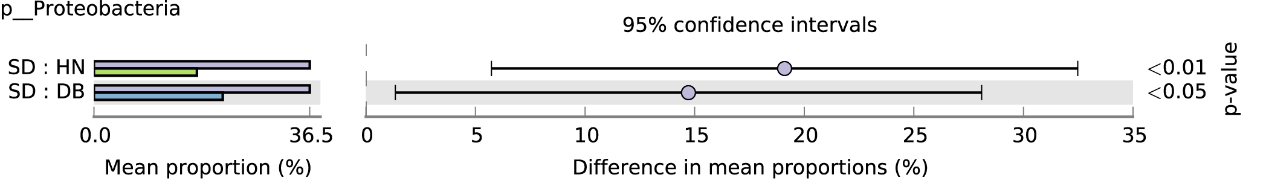

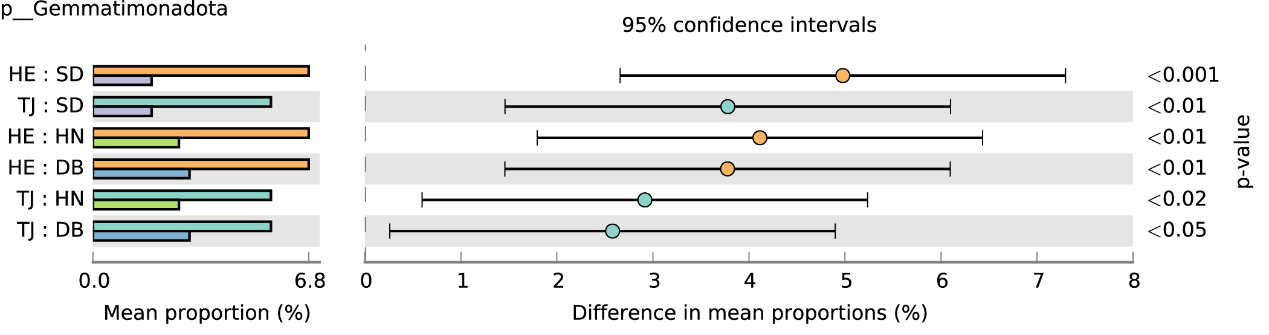

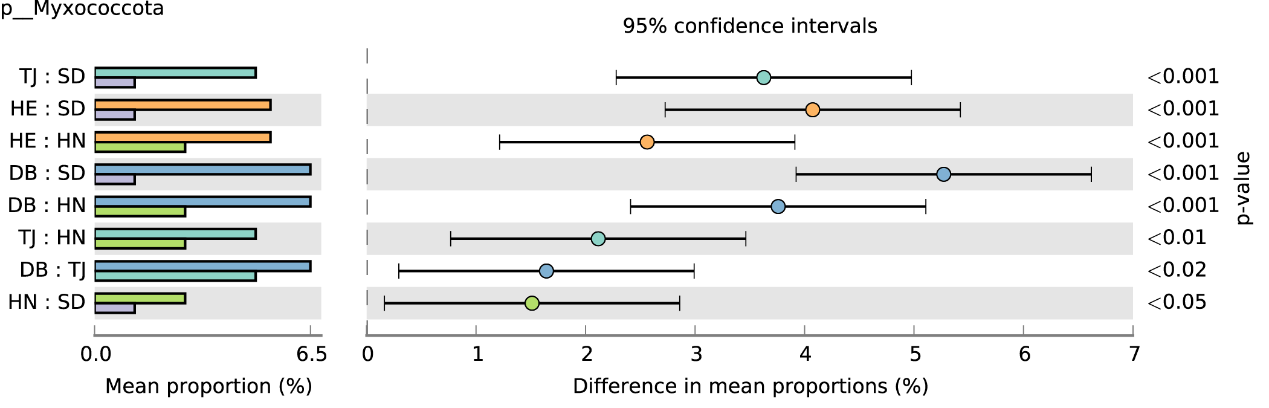

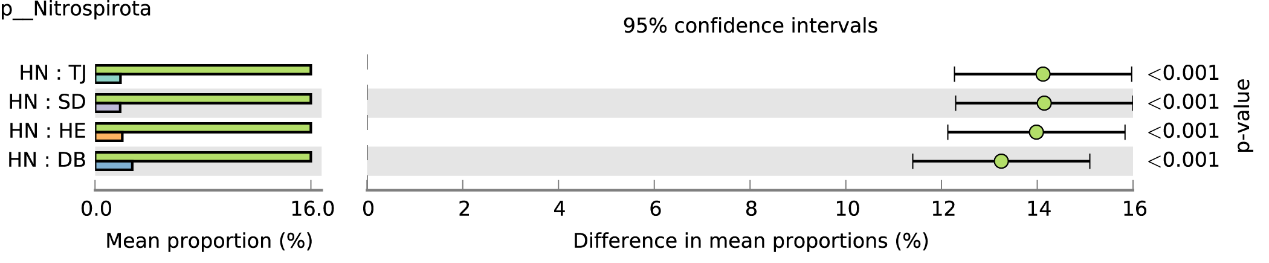

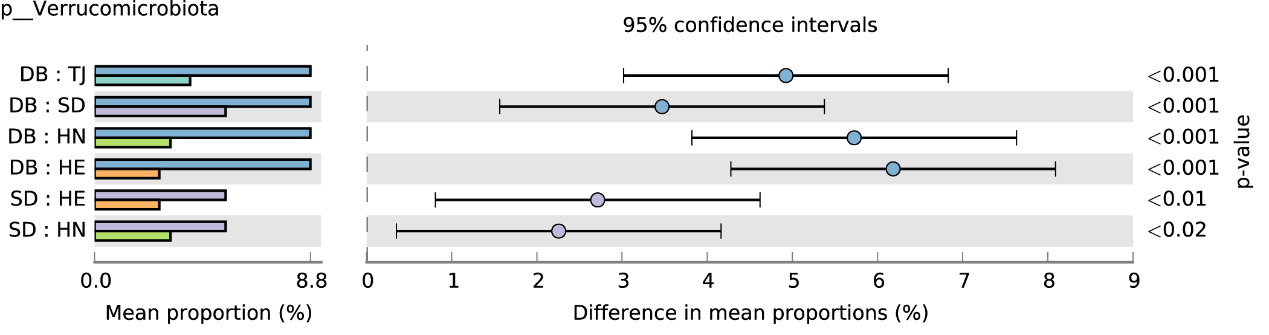
**

**Supplementary Figure 1** The bacterial taxa (phylum) showing significant differences of different paddy soils in 0-20 cm.

**
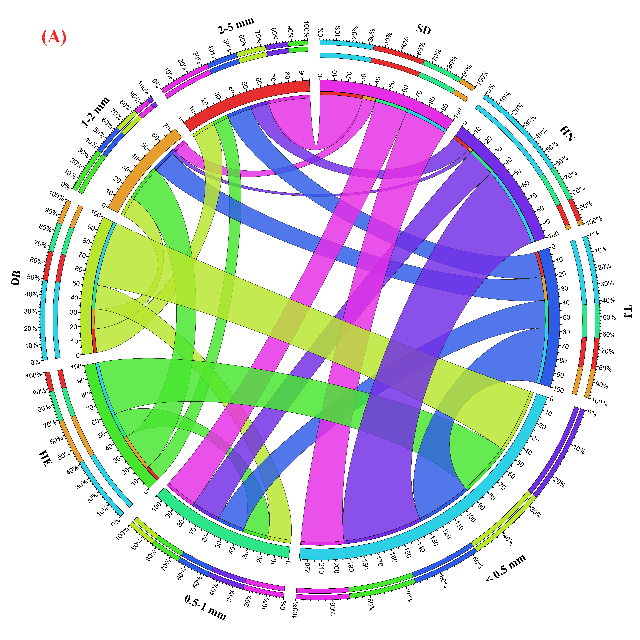

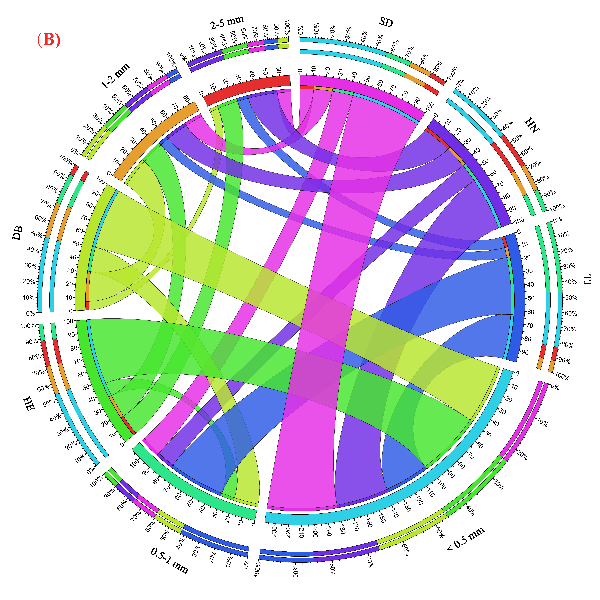








**

**Supplementary Figure 2** Size (A) in 0-20 cm, size (B) in 20-40 cm distribution of MPs of different paddy soils; violin plots showing the variance analysis of four sizes: <0.5 mm (C); 0.5-1 mm (D); 1-2 mm (E); 2-5 mm (F); Different capital letters indicate the significant difference among geographic position, and different lowercase letters indicate the significant differences between soil depths (*P*< 0.05) (n = 3).


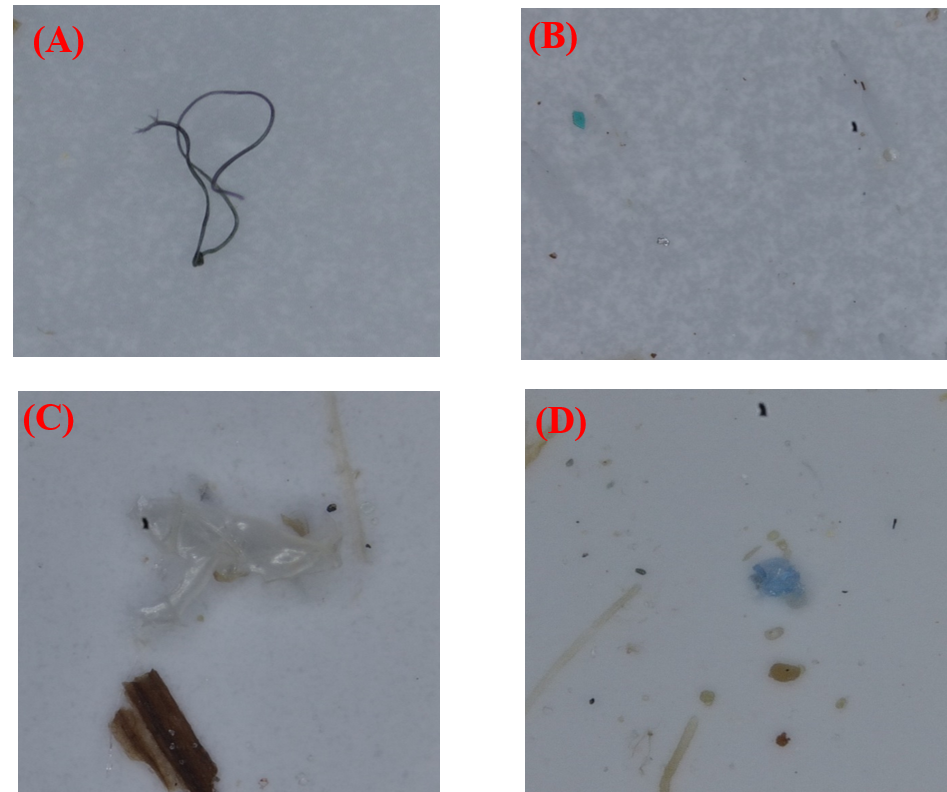


**Supplementary Figure 3** Microscopic images of four types of microplastics: fiber (A); pellet (B); film (C); fragment (D).

**


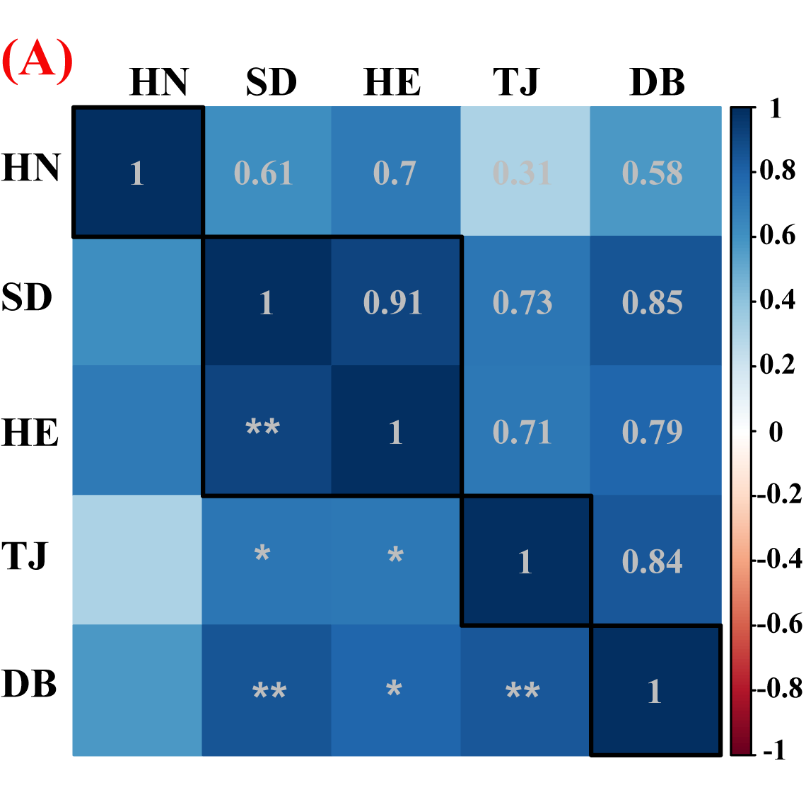
**

**Supplementary Figure 4** Mantel test based on Bray-Curtis distance, (A); the MDII index of paddy soils among different geographic positions, (B). Different lowercase letters indicate different levels (*P* < 0.05). Significant differences of paddy soils among different geographic positions were indicated by **P <* 0.05*, **P <* 0.01*, ***P <* 0.001*.*

**
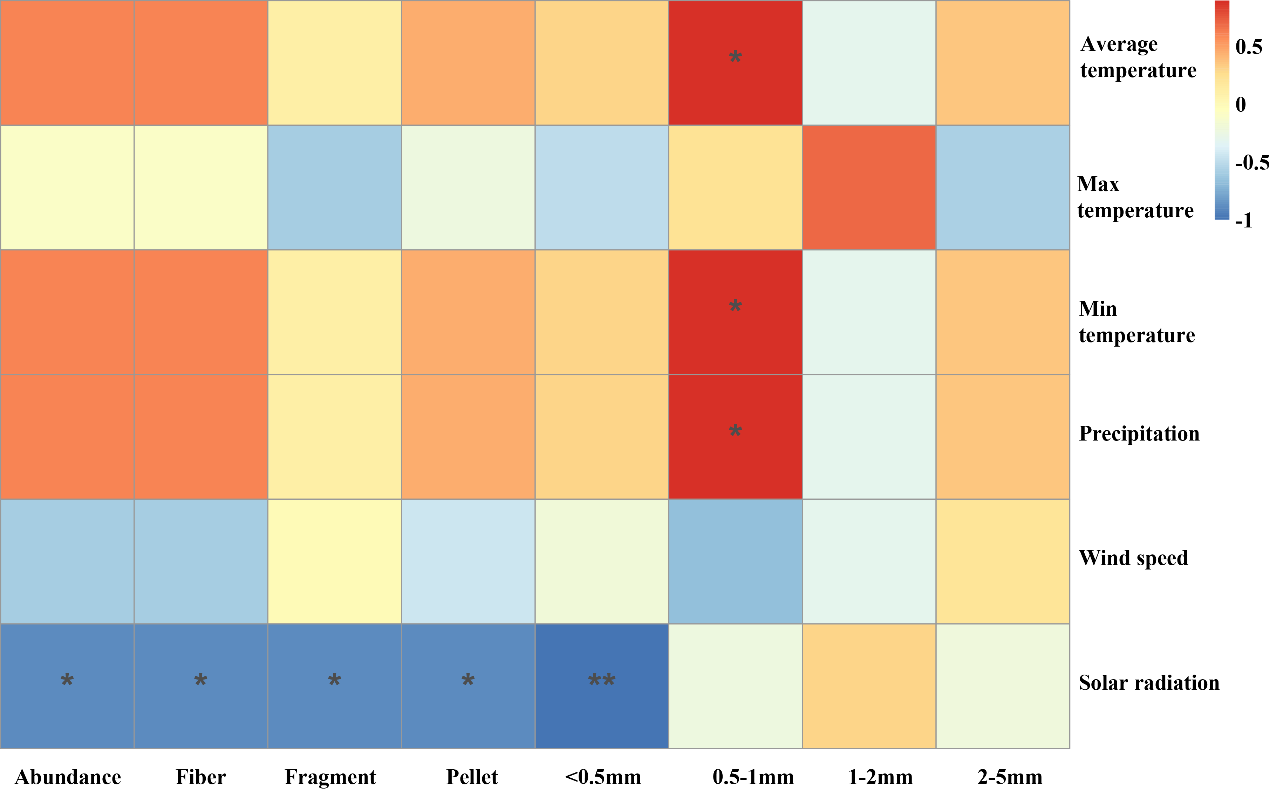
**

**Supplementary Figure 5** Linkage of main shape and size MP abundance with meteorological factors in 0-20 cm. Significant differences of distinct paddy soils were indicated by **P <* 0.05*, **P <* 0.01*, ***P <* 0.001.

**Supplementary Table 1** Two-way ANOVA of MP abundance of different paddy soil layers among different geographic positions

|  | **F** | **df** | **P** |
| --- | --- | --- | --- |
| **Type** | 42.396 | 4 | 0.000 |
| **Depth** | 25.673 | 1 | 0.000 |
| **Type*Depth** | 24.970 | 4 | 0.000 |
